# In the Company of Microbes: Ten Years of *Small Things Considered*

**DOI:** 10.3201/eid2402.171664

**Published:** 2018-02

**Authors:** Richard Danila

**Affiliations:** Minnesota Department of Health, Infectious Disease Epidemiology, Prevention and Control, St. Paul, Minnesot

**Keywords:** Elio Schaechter, Small Things Considered, blog, weblog, microbiology, archaea, bacteria, American Society for Microbiology, medical microbiology, historical reviews, microbiologist, teaching, technical essays typhus, retroviruses, fecal transplants, HIV vaccine, book review

In 2006, Elio Schaechter began a blog, titled *Small Things Considered*, about the microbial world for the American Society for Microbiology (ASM). The book, In the Company of Microbes: Ten Years of *Small Things Considered *(Figure), is a selected compilation of 70 of the more than 1,000 blog entries that were posted through 2015. Contributions from 33 writers in addition to Dr. Schaechter are included. These writers are past presidents of ASM and microbiologists from academic institutions around the world. The selections were chosen mostly because they were the authors’ personal or historical reflections on interactions with microbes. As such, these are aimed to be enjoyable reads, without bogging the reader down in obscurely technical scientific, often ephemeral, data.

**Figure:**
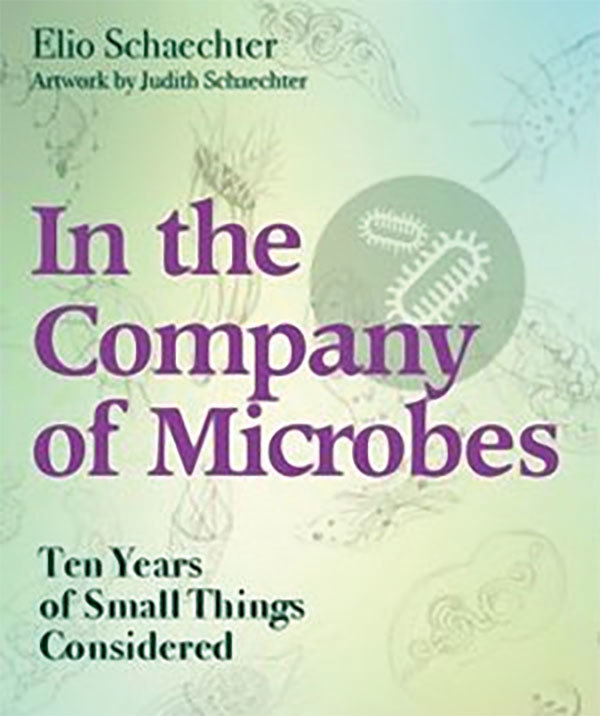
In the Company of Microbes: Ten Years of *Small Things Considered*

The pieces are divided into 7 sections, including personal musings of microbiologists, historical reviews, specifics of the microbial world, thoughts on being a microbiologist, teaching, and technical essays. The blog entries cover a breadth of information but have been selected because they emphasize the unusual or unexpected, often prompting a reaction of surprise, amusement, or curiosity to the reader. Interspersed throughout are Dr. Schaechter’s finely detailed questions, designed to provoke further thought and discussion. For example: “Given so many bacteria are intimately associated with animals and plants, why are so relatively few pathogenic?” “How does *Clostridium botulinum* benefit from making botulinum toxin?” “Is global warming likely to result in a significant net increase, decrease, or no change in the microbial biomass?” “Why are there so many species of microbes on earth?”

Because these are blogs, by design, they are very readable short selections; the book can be picked up and read and put down again and again without missing a beat. It certainly makes for good reading while traveling, sipping coffee, or during other quiet times.

Microbiologists shouldn’t be the only readers of this book. Anyone interested in the microbial world, including those in public health and clinical medicine, will appreciate many of the essays. There are some selections that those with less knowledge of molecular biology or microbial ecology will not understand and thus not benefit from reading. However, because this a collection of blog essays, a reader can simply move on to the next selection. Only a few readings relate closely to infectious diseases, including some on medical microbiology, typhus, retroviruses, fecal transplants, and HIV vaccines.

Modern microbiology is a wide field that has rapidly progressed during the past 50 years, encompassing the gamut from basic biology, biochemical engineering, applied mathematics, and theoretical physics. Centered on the ubiquity of the microbial world, these discussions touch everyone in the field and others interested in the disease effects of microbes on humans. This collection of well-written short essays is a notable accomplishment. 

